# The Overexpression of Twinkle Helicase Ameliorates the Progression of Cardiac Fibrosis and Heart Failure in Pressure Overload Model in Mice

**DOI:** 10.1371/journal.pone.0067642

**Published:** 2013-06-28

**Authors:** Atsushi Tanaka, Tomomi Ide, Takeo Fujino, Ken Onitsuka, Masataka Ikeda, Takako Takehara, Yuko Hata, Emil Ylikallio, Henna Tyynismaa, Anu Suomalainen, Kenji Sunagawa

**Affiliations:** 1 Department of Cardiovascular Medicine, Graduate School of Medical Sciences, Kyushu University, Fukuoka, Japan; 2 Research Programs Unit, Molecular Neurology, Biomedicum-Helsinki, University of Helsinki, Helsinki, Finland; University of Kansas Medical Center, United States of America

## Abstract

Myocardial mitochondrial DNA (mtDNA) copy number decreases in heart failure. In post-myocardial infarction mice, increasing mtDNA copy number by overexpressing mitochondrial transcription factor attenuates mtDNA deficiency and ameliorates pathological remodeling thereby markedly improving survival. However, the functional significance of increased mtDNA copy number in hypertensive heart disease remains unknown. We addressed this question using transgenic mice that overexpress Twinkle helicase (Twinkle; Tg), the mtDNA helicase, and examined whether Twinkle overexpression protects the heart from left ventricular (LV) remodeling and failure after pressure overload created by transverse aortic constriction (TAC). Twinkle overexpression increased mtDNA copy number by 2.2±0.1-fold. Heart weight, LV diastolic volume and wall thickness were comparable between Tg and wild type littermates (WT) at 28 days after TAC operation. LV end-diastolic pressure increased in WT after TAC (8.6±2.8 mmHg), and this increase was attenuated in Tg (4.6±2.6 mmHg). Impaired LV fractional shortening after TAC operation was also suppressed in Tg, as measured by echocardiography (WT: 16.2±7.2% vs Tg: 20.7±6.2%). These LV functional improvements were accompanied by a decrease in interstitial fibrosis (WT: 10.6±1.1% vs Tg: 3.0±0.6%). In *in vitro* studies, overexpressing Twinkle using an adenovirus vector in cultured cardiac fibroblasts significantly suppressed mRNA of collagen 1a, collagen 3a and connective tissue growth factor, and angiotensin II-induced transforming growth factor β1 expression. The findings suggest that Twinkle overexpression prevents LV function deterioration. In conclusion, Twinkle overexpression increases mtDNA copy number and ameliorates the progression of LV fibrosis and heart failure in a mouse pressure overload model. Increasing mtDNA copy number by Twinkle overexpression could be a novel therapeutic strategy for hypertensive heart disease.

## Introduction

Heart failure is the end-stage of various heart conditions and diseases, and has become a major public health problem in most countries. [1], [2]. Hypertension is a common risk factor for heart failure, followed closely by antecedent myocardial infarction. Seventy-five percent of heart failure cases have antecedent hypertension [1]. Hypertension affects approximately one billion people worldwide [3]. Sustained cardiac pressure overload induces cellular, molecular and morphologic remodeling and maladaptations contributing to progressive cardiac dysfunction and heart failure [4]. However, except antihypertensive drugs, there is no known effective medical treatment to attenuate pressure overload-induced cardiac remodeling. New therapeutic strategies to prevent maladaptive remodeling and subsequent progression to heart failure in hypertensive heart disease are highly desirable.

Mitochondrial dysfunction has been reported in various forms of heart failure. Especially, mitochondrial DNA (mtDNA) copy number is decreased in the heart of post-myocardial infarction mice [5] and pressure overload models [6]. In humans, Karamanlidis et al. [7] demonstrated that mitochondrial biogenesis is severely impaired in myocardial tissues collected from patients with end-stage heart failure of various etiologies. In a mouse post-myocardial infarction model, overexpression of mitochondrial transcription factor A by a transgenic approach ameliorated the decrease in mtDNA copy number and pathological remodeling, dramatically improving survival [8]. These findings indicate that increasing mtDNA copy number attenuates cardiac pathological remodeling and failure. However, the functional significance of increased mtDNA copy number under pressure overload condition has not been established.

In this study, we addressed this question using transgenic mice that overexpress Twinkle, the mtDNA helicase. Previous study showed that systemic overexpression of Twinkle increases mtDNA copy number in muscle and heart up to 3-fold of control levels, more than any other factors reported to date [9]. Twinkle is known to co-localize with mtDNA in mitochondrial nucleoids that are stable assemblies of nucleoproteins and mtDNA. Twinkle displays 5′ to 3′ DNA helicase activity *in vitro*, supporting its role in unwinding the mtDNA replication fork [10]. Dominant mutations of Twinkle are associated with progressive external ophthalmoplegia with multiple mtDNA deletions [11]. Reduced Twinkle expression by RNA interference also mediates a rapid drop in mtDNA copy number, supporting the *in vivo* results [9]. These data demonstrate that Twinkle is essential for mtDNA maintenance, and that it may be a key regulator of mtDNA copy number in mammals [9], [12].

In a pilot study, we have confirmed that overexpressing Twinkle in mice by a transgenic approach inhibits cardiac remodeling and improves survival after experimental myocardial infarction (unpublished data). However, the functional significance of increased Twinkle in pressure overload-induced cardiac remodeling remains unclear. In this study, we examined whether Twinkle overexpression protects the heart from left ventricular (LV) remodeling and failure in a mouse pressure overload model created by transverse aortic constriction (TAC).

## Materials and Methods

### Ethics Statement

All procedures and animal care were approved by the Committee on Ethics of Animal Experiment, Kyushu University Graduate School of Medical and Pharmaceutical Sciences (Permit number: A22–075), and performed in accordance with the Guideline for Animal Experiment of Kyushu University, and the Guide for the Care and Use of Laboratory Animals published by the US National Institutes of Health (NIH Publication No. 85-23, revised 1996).

### Animal Experiments

We utilized transgenic mice that overexpress mouse Twinkle helicase (Tg) as described previously [9]. The animals were kept in 12-hour light-dark cycle and had access to food and water *ad libitum*. Ten week-old male Tg and wild type littermates (WT) underwent TAC as described previously with a slight modification [13]. Briefly, mice were anesthetized with sodium pentobarbital (35 mg/kg intraperitoneally) and intubated endotracheally. The chest was opened and the aortic arch was identified after blunt dissection through the intercostal muscles. A 8-0 silk suture was placed around the transverse aorta and tied with a 26-gauge blunt needle, which was immediately removed. We used a 26-gauge and tied the suture as tightly as possible to create similar degree of pressure gradient in all mice. Sham-operated mice underwent a similar surgical procedure without aortic constriction. Animals were anesthetized and euthanized 28 days after TAC for physiological, biochemical and histological studies.

A group of investigators performed tail clippings, and used the tissue samples in genotyping using polymerase chain reaction (PCR). Another group of investigators who were not informed of the genotyping results performed TAC. Animals were identified by numeric codes and assigned to experimental groups.

### PCR Analyses

We quantified mtDNA copy number and mRNA expression in the heart and cardiac fibroblasts by real-time PCR analyses as described previously [14], [15]. Heart samples were homogenized, and total DNA was extracted by DNeasy Blood & Tissue Kit (Qiagen). Total DNA was treated with BamHI (Takara) for 6 hours and used in quantitative PCR to estimate the relative quantity of mtDNA. The 30-µl PCR mixture contained 5 ng of total DNA and 12 pmol each of the primers (5′-TGTAAGCCGGACTGCTAATG-3′ and 3′-AGCTGGAGCCGTAATTACAG-5′ for mtDNA). As an internal standard, ribosomal protein L27 (RPL27) gene was amplified in a 30-µl reaction mixture containing 5 ng of total DNA and 12 pmol each of the primers (5′-CCTCATGCCCACAAGGTACTC-3′ and 3′-TCGCTCCTCAAACTTGACC-5′). The amount of mtDNA was adjusted to the amount of genomic DNA. All reactions were performed with SYBR Premix Ex Taq II (Takara) and Applied Biosystems 7500 Real-Time PCR system (Applied Biosystems) according to the manufacture’s protocol.

Heart samples for RNA analysis were stored in RNAlater (Ambion). After homogenization, total RNA was extracted with RNeasy Mini Kit (Qiagen). After reverse transcription with ReverTra Ace qPCR RT kit (Toyobo), the relative amount of cDNA was quantified using a 30-µl reaction mixture containing 10 ng of total cDNA and 12 pmol each of the primers [5′-GACTGGCAACCTCAAGAAGG-3′ and 3′-GACTGTCTTGCCCCAAGTTC-5′ for collagen 1a (COL1a), 5′-CTGTAACATGGAAACTGGGGAAA-3′ and 3′-CCATAGCTGAACTGAAAACCACC-5′ for collagen 3a (COL3a), and 5′-TGCAGACTGGAGAAGCAGAG-3′ and 3′-CGATTTTAGGTGTCCGGATG-5′ for connective tissue growth factor (CTGF)]. We used hypoxanthine guanine phosphoribosyl transferase (HPRT) gene as an internal standard (primers: 5′-CTGGTGAAAAGGACCTCTCG-3′ and 3′-AACTTGCGCTCATCTTAGGC-5′). In *in vitro* analyses, the relative amount of cDNA was quantified using a 30-µl reaction mixture containing 10 ng of total cDNA and 12 pmol each of the primers (5′-CATTGCTGTCCCGTGCAGA-3′ and 3′-AGGTAACGCCAGGAATTGTTGCTA-5′) for transforming growth factor β1 (TGF-β1). We used ribosomal protein S18 (18S) gene as an internal standard (primers: 5′-AAGTTTCAGCACATCCTGCGAGTA-3′ and 3′-TTGGTGAGGTCAATGTCTGCTTTC-5′).

### Mitochondrial Isolation and Blue Native Gel Electrophoresis

We measured mitochondrial protein and enzyme activity as described previously [16], [17]. Hearts were homogenized in ice-cold HIM buffer (200 mM mannitol, 70 mM sucrose, 10 mM HEPES, 1 mM EGTA, adjusted to pH 7.5 with KOH) using a zero-clearance Teﬂon pestle, and centrifuged at 600×*g* for 20 minutes. The supernatant was further centrifuged at 600×*g* for 20 minutes and at 10000×*g* for 10 minutes. The resulting mitochondrial pellet was washed with HIM buffer and centrifuged again at 10000×*g* for 10 minutes. The pellet was resuspended in phosphate-buffered saline containing a protease inhibitor cocktail, and the protein concentration was determined. Native gradient gels (5–12%) were casted and run according to the protocol described previously [18]. Mouse monoclonal antibodies against complexes I (MS111, 1∶1000), II (MS204, 1∶10000), and III (MS302, 1∶1000) from Mitosciences were diluted in Tris-buffered saline containing 0.1% Tween and 5% milk. Equal amount of mitochondrial protein extract (2.5 µg) from each group was loaded per well. We normalized complexes I and III protein levels and complex I activity against those of complex II.

### Echocardiographic and Hemodynamic Measurements

We performed *in vivo* analyses of mice as described previously [8], [19]. On day 28 after TAC surgery, echocardiographic studies were performed under anesthesia with a mixture of medetomidine (0.3 mg/kg), midazolam (4 mg/kg) and butorphanol tartrate (5 mg/kg), and spontaneous respiration. A 2D parasternal short-axis view of the LV was obtained at the level of the papillary muscles by applying the transducer lightly to the mid-upper left anterior chest wall. After ensuring that the image was on axis (based on roundness of the LV cavity), 2D targeted M-mode tracings were recorded at a paper speed of 50 mm/s. Anterior, posterior end diastolic wall thickness and LV internal dimensions were measured. While under anesthesia, a 1.4 Fr micromanometer-tipped catheter (Millar Instruments) was inserted into the right carotid artery and advanced into the LV to measure pressures for the assessment of aortic pressure and LV end diastolic pressure.

### Histopathological Studies

After *in vivo* echocardiographic and hemodynamic studies, the heart was excised and weighed, and dissected into the right and left ventricles, including the septum. The heart tissues were fixed in 6% formaldehyde, embedded in paraffin, and cut into 5 µm thick sections. Sections were stained with hematoxylin-eosin and Masson’s trichrome for assessments of myocyte cross-sectional area and collagen volume fraction [13]. To measure the myocyte cross-sectional area, each section was photographed under a microscope (DMD108, Leica Microsystems) at a final magnification of 200×. The profiles of 30 to 40 myocytes cut in cross-sections were traced manually and digitized. The digitized profiles were transferred to a personal computer that calculated the area. Three to 4 fields were randomly selected from 3 coronal sections of each heart. Thus, 100 to 200 myocytes were measured for each animal, and the mean myocyte cross-sectional area was calculated. Collagen volume fraction was measured in 6 fields randomly selected from each coronal section (basal, mid and apical sections) in each animal. Each field was photographed under a microscope at a final magnification of 200×, and subjected to color threshold analysis. Collagen volume fraction for the heart was calculated as the sum of all connective tissue areas divided by the sum of all connective tissue and muscle areas in all fields. Collagen surrounding intramyocardial coronary arteries was excluded from analysis.

### Plasmid Construction

Small-interfering RNA (siRNA) targeting rat Twinkle helicase (si-rTwinkle) was synthesized by Takara (Shiga, Japan). The si-rTwinkle gene was sub-cloned into unique BamHI and HindIII sites of pBAsicDNA Vector (Takara, pBAsi-rTwinkle). The full length human Twinkle helicase complimentary DNA (cDNA, hTwinkle) was amplified by PCR with primers containing XbaI and HindIII sites extracted from the placenta human cDNA library. The cDNA library was provided by the Department of Clinical Chemistry and Laboratory Medicine, Graduate School of Medical Sciences, Kyushu University. The PCR product was sub-cloned into distinctive XbaI and HindII sites of the pcDNA3.1 Expression Vector (Invitrogen, pcDNAhTwinkle). The pBAsi-rTwinkle and pcDNAhTwinkle were amplified, sequenced and used for constructing adenovirus [20].

### Adenovirus Transduction

Replication-deficient recombinant adenovirus vectors containing hTwinkle (AxCAhTwinkle), si-rTwinkle (AxCAsi-rTwinkle) or *Escherichia* coli Lac*Z* cDNA (AxCALacZ) were constructed using Adenovirus Expression Vector Kit Ver. 2 (Takara) according to manufacturer’s protocol. Adenoviruses were amplified in human embryonic kidney cell line (HEK-293, RIKEN BIORESOURCE CENTER, Cell No. RCB1637) purified with the Adeno-X Maxi purification Kit (Clontech) and then titrated with the Adeno-X Rapid Titer kit (Clontech). The efficiency of virus infection was >95%, as measured by β-gal staining.

### *In vitro* Experiments

Primary culture of neonatal rat cardiac fibroblasts was prepared from the ventricles of neonatal Sprague-Dawley rats as described previously [15], [21]. Briefly, neonatal rats were euthanized by decapitation under anesthesia with isoflurane, after which the hearts were rapidly excised and digested. Anesthesia depth was monitored by limb withdrawal in response to toe pinching. After digesting the myocardial tissue with trypsin (Wako) and collagenase type 2 (Worthington), cells were suspended in Dulbecco’s Modified Eagle’s Medium (DMEM, Sigma-Aldrich) containing 10% fetal bovine serum (Thermo SCIENTIFIC), penicillin (Invitrogen) and streptomycin (Invitrogen), and plated in 100 mm culture dishes for 70 minutes to remove non-adherent cardiac myocytes. Adherent cardiac fibroblasts were maintained at 37°C in humidified air with 5% CO_2_. Considering the possibility that cardiac fibroblasts may lose the original characteristics after prolonged culture, cells were used within 2 passages in all experiments. Cells were infected with AxCAhTwinkle, AxCAsi-rTwinkle or AxCALacZ (multiplicity of infection; 1) in serum-free DMEM for 1 hour, and cultured for another 72 hours in DMEM containing 5% fetal bovine serum. Then the cells were stimulated with angiotensin II (AngII, Sigma-Aldrich, 1 µM) for 24 hours, and collected for mRNA analyses.

### Statistical Analysis

All data were expressed as mean ± SEM. Between-group comparison of means was performed by one-way analysis of variance followed by Bonferroni’s post-hoc test. A *P*-value less than 0.05 was considered to be statistically significant.

## Results

### mtDNA Copy Number and Mitochondrial Enzyme Activity

**Figure 1:**
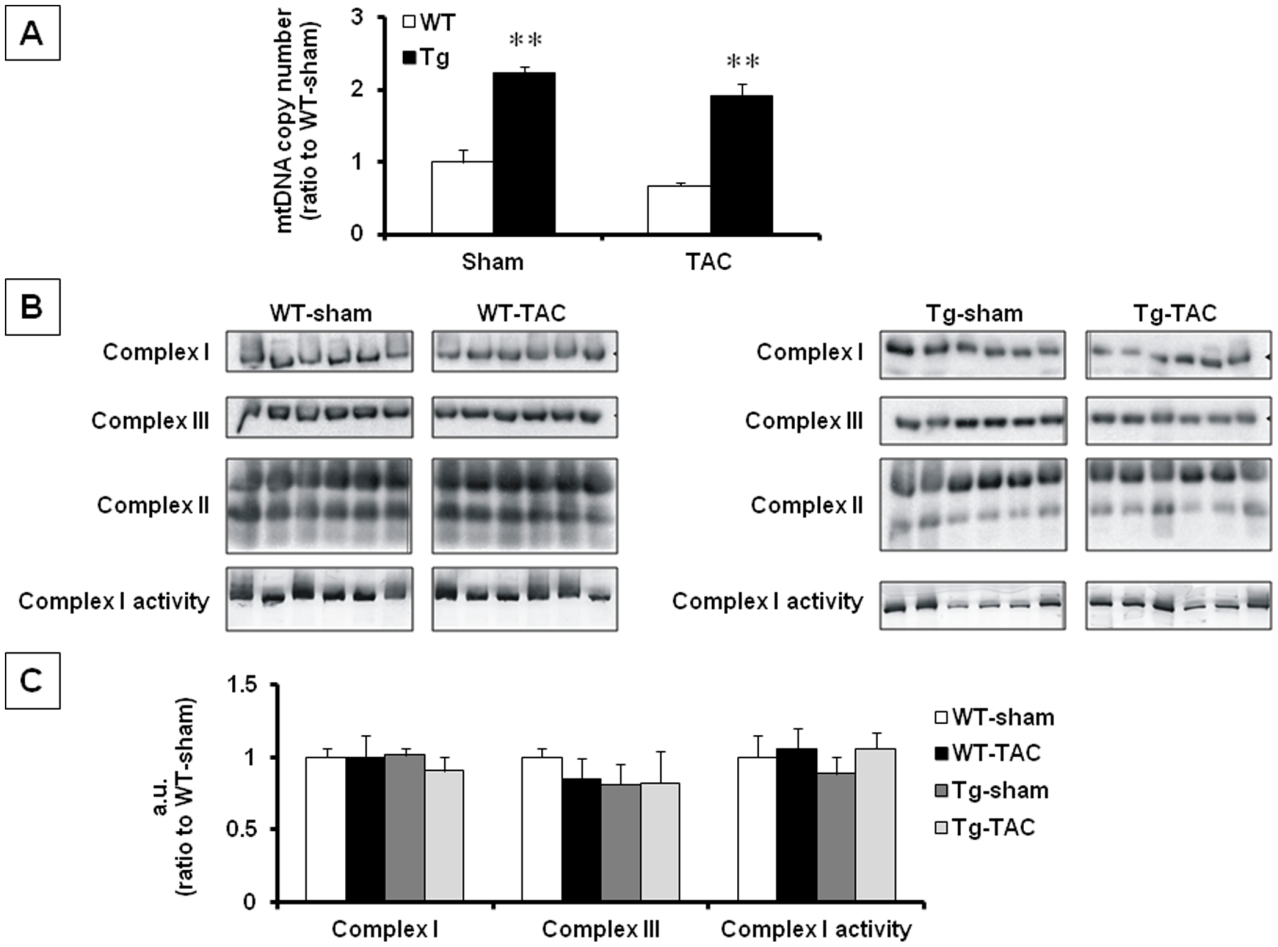
mtDNA copy number and mitochondrial enzyme activity. A. mtDNA copy number after sham or TAC operation, quantified by real-time PCR relative to nuclear genome (RPL27 gene). B. Mitochondrial protein amount and enzyme activity. Mitochondrial protein amount (complexes I, II and III) and enzyme activity (complex I) of WT (left panels) and Tg (right panels), n = 6 each, were analyzed by blue native gel electrophoresis. C. The signals for complexes I, III and complex I activity were normalized against those of complex II. Values are mean ± SEM. Data are presented as ratio to WT-sham. **; *P*<0.01 vs WT-sham.

We first examined the mitochondrial characteristics in Tg and WT that were sham-operated or underwent TAC. mtDNA copy number increased significantly in Tg compared to WT, both in sham-operated (2.2-fold, *P*<0.01) and TAC groups (2.9-fold, *P*<0.01). mtDNA copy number tended to decrease on day 28 after TAC in both WT (*P* = 0.07 WT-TAC vs. WT-sham) and Tg (*P* = 0.11, Tg-TAC vs. Tg-sham), although the differences were not significant in both groups (Figure 1A). Mitochondrial complexes I and III protein levels and mitochondrial complex I activity were normalized against those of complex II which is entirely encoded by the nucleus [16]. Both mitochondrial protein levels and activities were not affected by Twinkle overexpression, consistent with previous report [16], and were not altered by TAC (Figure 1B and C).

### Cardiac Function and Structure

Table 1 shows the hemodynamic data and Table 2 shows the organ weights on day 28 after TAC operation. TAC increased heart weight and LV weight in both WT and Tg, although there was no significant differences between Tg-TAC and WT-TAC. TAC also increased aortic pressure, again with no difference between Tg-TAC and WT-TAC. Importantly, Twinkle overexpression significantly inhibited the increase in LV end-diastolic pressure caused by TAC-induced pressure overload (*P*<0.05, Tg-TAC vs. WT-TAC).

**Table 1:** Hemodynamic data.

	WT-sham	Tg-sham	WT-TAC	Tg-TAC
	(n = 8)	(n = 3)	(n = 11)	(n = 11)
HR (bpm)	522±21	506±10	521±32	535±37
Peak BP (mmHg)	101±7	111±10	172±24**	167±21**
Mean BP (mmHg)	86±3	77±12	116±6**	111±11**
LVEDP (mmHg)	1.4±1.4	1.0±0.5	8.6±2.8**	4.6±2.6* †

HR; heart rate, BP; blood pressure, LVEDP; LV end-diastolic pressure.

** ; *P*<0.01,

* ; *P*<0.05 vs WT-sham,

† ; *P*<0.05 vs WT-TAC.

**Table 2:** Organ weight data.

	WT-sham	Tg-sham	WT-TAC	Tg-TAC
	(n = 17)	(n = 12)	(n = 17)	(n = 16)
Body wt (g)	29.0±2.9	28.3±1.8	27.3±1.6*	27.5±1.5*
Heart wt/body wt (mg/g)	4.9±0.4	4.9±0.7	6.8±0.9*	6.4±0.7*
LV wt/body wt (mg/g)	3.2±0.3	3.2±0.3	5.0±0.9*	4.5±0.6*

* ; *P*<0.05 vs WT-sham.

**Figure 2:**
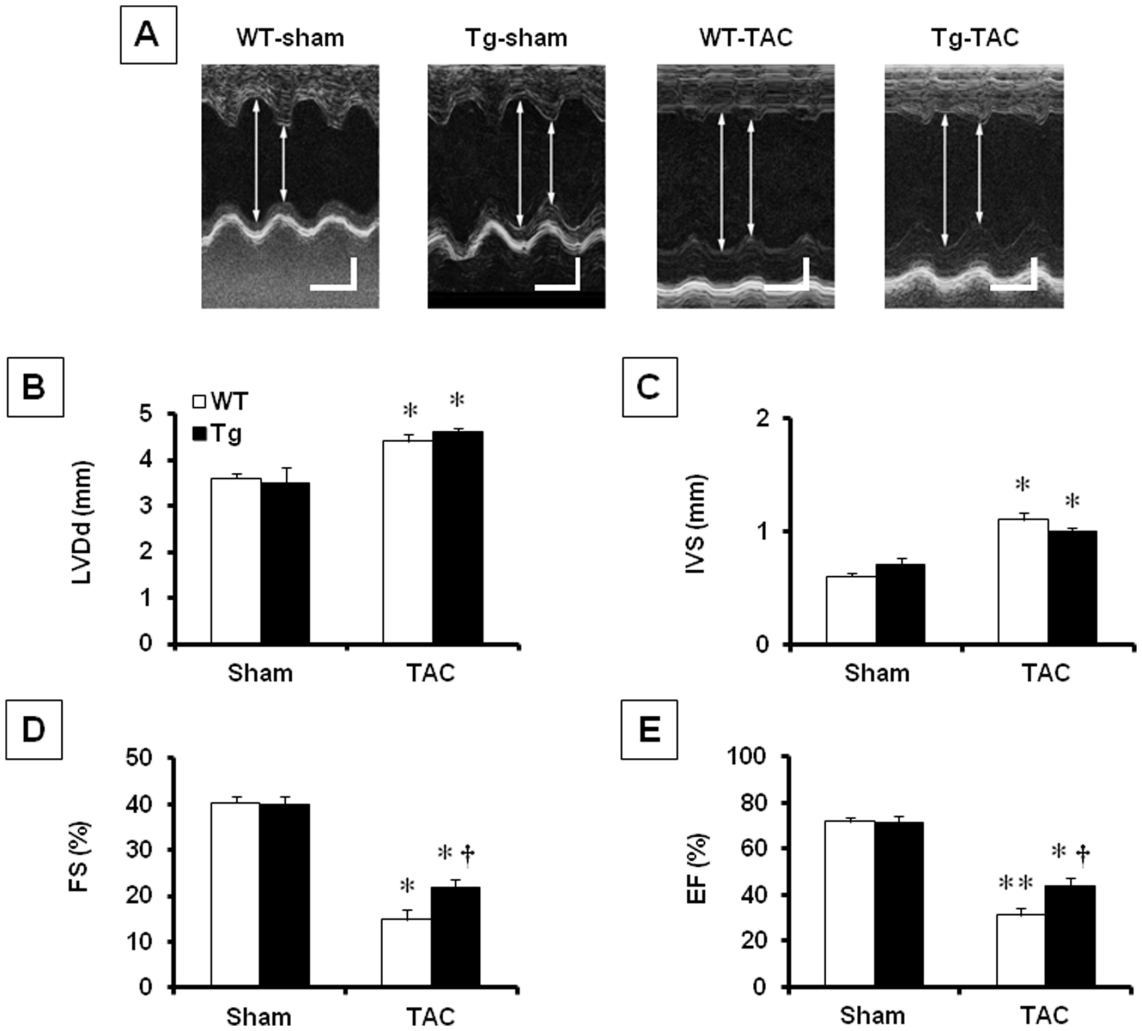
Echocardiographic analyses. A. Representative M-mode echocardiograms obtained from WT-sham, Tg-sham, WT-TAC, and Tg-TAC. Arrows indicate LV end-diastolic and end-systolic diameter. Scale bar = 100 msec (horizontal) and 1 mm (vertical). B-E. Summary data for echocardiographic measurements in 4 groups of animals: LV end-diastolic diameter (B), interventricular septal thickness (C), percent fractional shortening (D) and percent left ventricular ejection fraction (E). Values are mean ± SEM. *; *P*<0.05 vs WT-sham, **^†^**; *P*<0.05 vs WT-TAC. LVDd; LV end-diastolic diameter, IVS; interventricular septum, FS; fractional shortening, EF; ejection fraction.

Echocardiographic study showed enlarged LV end-diastolic dimension after TAC operation in both Tg and WT, with no significant differences between Tg-TAC and WT-TAC (Figure 2A and B). There was also no difference in LV wall thickness between Tg and WT (Figure 2C). However, fractional shortening decreased by approximately 60% in WT-TAC compared with WT-sham but by only 49% in Tg-TAC (*P*<0.05, Tg-TAC vs. WT-TAC). Similar results were observed for ejection fraction. These results suggest relatively preserved LV function in Tg-TAC (Figure 2D and E). We also found a tendency of LV dysfunction attenuation in Tg-TAC mice on day 14 after TAC operation (Figure S1).

**Figure 3:**
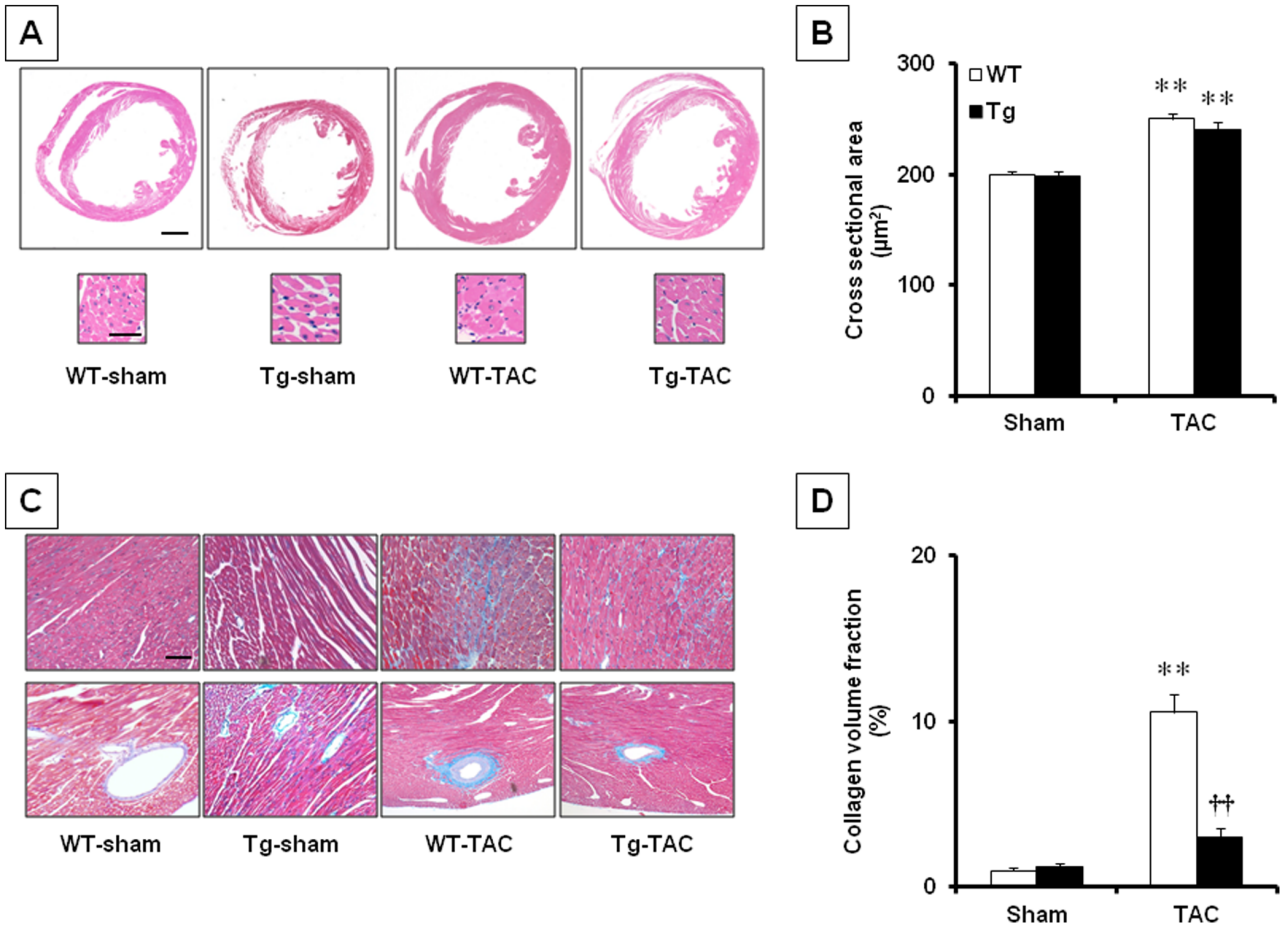
Histopathological analyses. A. Representative photomicrographs of hematoxylin-eosin-stained LV cross-sections obtained from 4 groups of animals. Scale bar = 1 mm (upper sections) and 50 µm (lower sections). B. Myocyte cross-sectional area in WT-sham, Tg-sham, WT-TAC and Tg-TAC. C. Representative photomicrographs of Masson’s trichrome-stained LV cross-sections obtained from 4 groups of animals. Scale bar = 50 µm. D. Collagen volume fraction in WT-sham, TG-sham, WT-TAC, and TG-TAC. Values are mean ± SEM. **; *P*<0.01 vs WT-sham. **^††^**; *P*<0.01 vs WT-TAC.

In histological analyses, we assessed the cross-sectional area of cardiac myocyte as an index of hypertrophy. Consistent with the data of LV weight and echocardiographic wall thickness, the cross-sectional area increased markedly after TAC operation in both Tg and WT, although there was no significant difference between WT-TAC and Tg-TAC (Figure 3A and B). Next we investigated the progression of fibrosis in myocardium. We found marked interstitial fibrosis in the myocardium, on day 28 after TAC operation. Twinkle overexpression significantly suppressed the TAC-induced increase in fibrosis (*P*<0.01, Tg-TAC vs. WT-TAC; Figure 3C and D). Meanwhile, expressions of COL1a, COL3a and CTGF, which are commonly used markers of fibrosis, tended to increase in WT-TAC on day 28 after TAC operation, but Twinkle overexpression tended to inhibit these increase (Figure S2), supporting our histological result of the inhibition of cardiac fibrosis.

### *In vitro* Experiments

**Figure 4:**
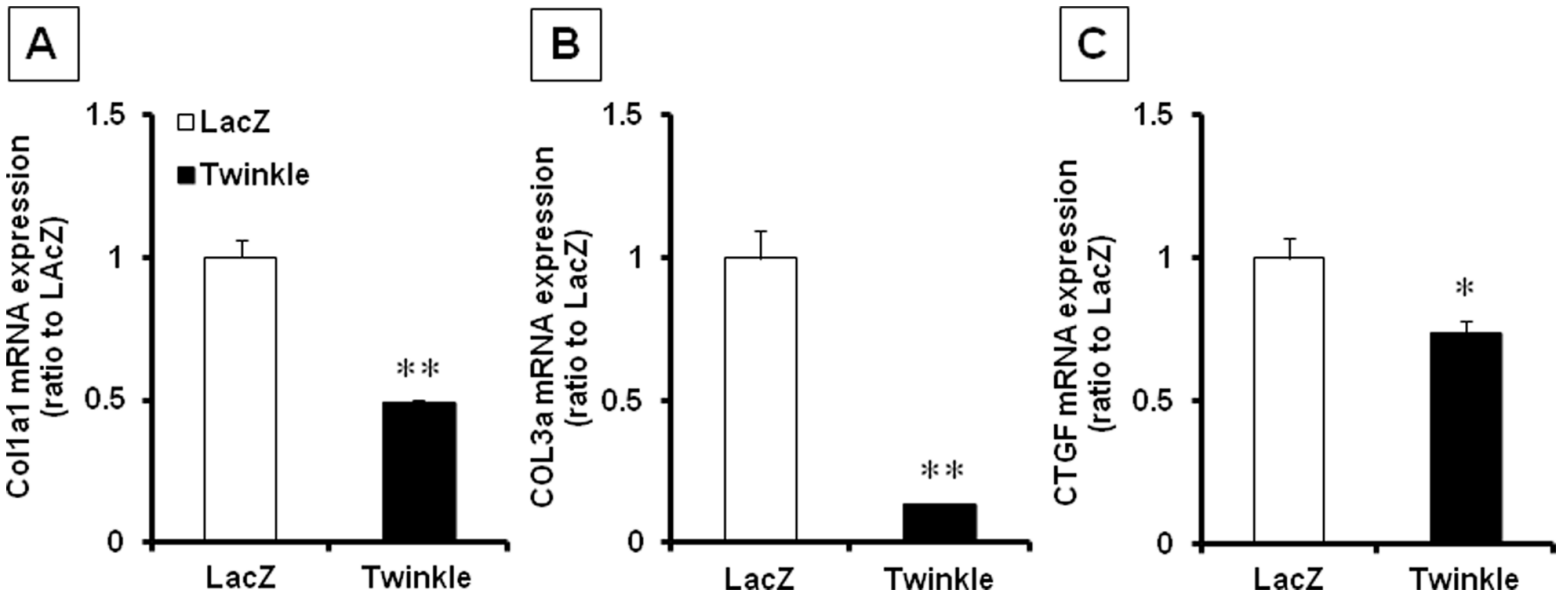
Effects of the upregulation of Twinkle on fibrosis signaling. A-C. mRNA expression of COL1a (A), COL3a (B), and CTGF (C), quantified by real-time PCR relative to housekeeping gene (18S gene) in neonatal rat cardiac fibroblast. Cells were preinfected with AxCAhTwinkle (Twinkle) or AxCALacZ (LacZ) for 72 hours. Values are mean ± SEM. Data are presented as ratio to LacZ. **; *P*<0.01, *; *P*<0.05 vs LacZ.

**Figure 5:**
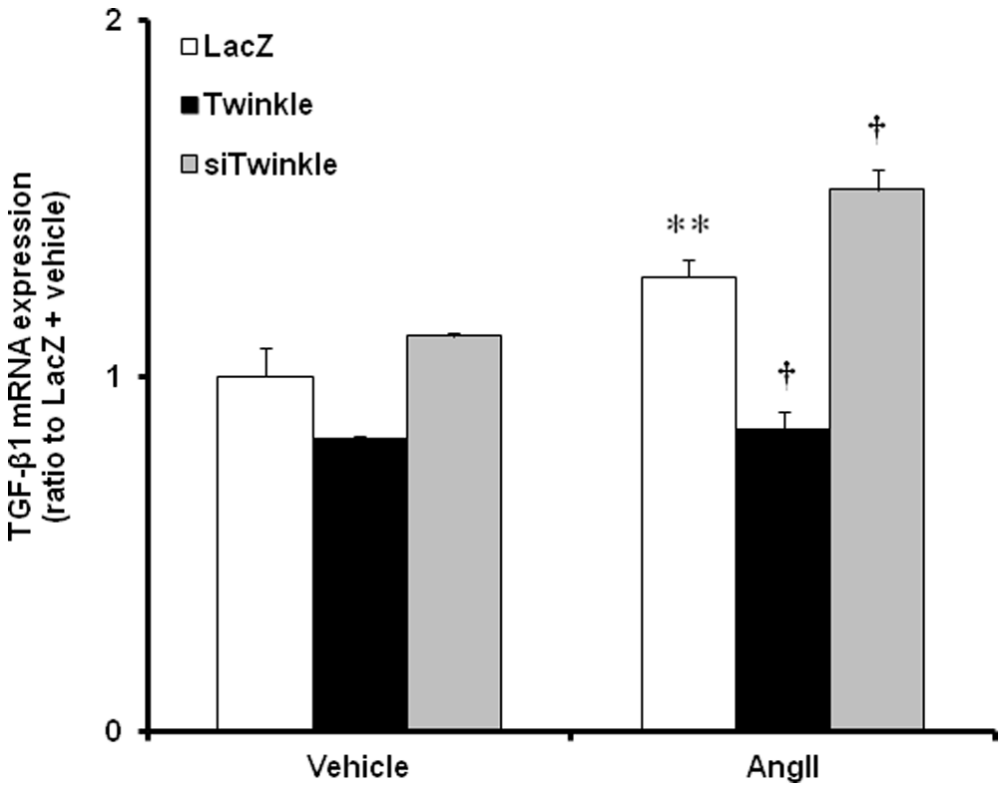
Effects of the upregulation or donwregulation of Twinkle on TGF-β1 mRNA expression. TGF-β1 expression in cardiac fibroblasts stimulated with AngII (1 µM) for 24 hours, quantified by real-time PCR relative to housekeeping gene (18S gene). Cells were preinfected with AxCAhTwinkle (Twinkle), AxCAsi-rTwinkle (siTwinkle) or AxCALacZ (LacZ). Values are mean ± SEM. Data are presented as ratio to LacZ-vehicle. **; *P*<0.01 vs LacZ-vehicle, **^†^**; *P*<0.05 vs LacZ-AngII.

In order to confirm the alteration of mRNA in fibroblast specifically, we checked profibrogenic signals in cardiac fibroblast isolated from neonatal rat heart. We found significant suppression in all 3 mRNAs, COL1a, COL3a, and CTGF (Figure 4). To examine the mechanism by which Twinkle overexpression inhibits cardiac fibrosis *in vitro*, we prepared rat neonatal cardiac fibroblasts and stimulated with AngII for 24 hours. AngII increased TGF-β1 mRNA expression, which is a key regulator of fibrosis [22], [23]. Twinkle overexpression by adenovirus vector suppressed TGF-β1 expression in cardiac fibroblasts, compared with LacZ overexpression. In contrast, downregulation of Twinkle by siRNA, which inhibited Twinkle mRNA by 35% (Figure S3), exacerbated TGF-β1 expression (Figure 5). These findings suggest that Twinkle overexpression inhibits cardiac fibrosis by suppressing profibrogenic signals in the myocardium.

## Discussion

Hypertension is a major public health problem, affecting approximately one billion people worldwide [3]. Sustained pressure overload causes hypertrophic changes in the myocardium. While these changes may represent adaptive remodeling in the early phase, they eventually progress to maladaptive remodeling and exacerbate heart failure. No therapeutic options are currently available to prevent the progression to maladaptive remodeling for hypertensive heart disease. We report for the first time that Twinkle overexpression ameliorates cardiac fibrosis and heart failure in a mouse pressure overload model. In this study, Twinkle overexpression did not inhibit myocardial hypertrophy (adaptive remodeling), but prevented the pathological fibrotic change and progression of heart failure (maladaptive remodeling). We propose a new potential therapeutic concept that increasing Twinkle could be beneficial to prevent heart failure cause by prolonged pressure overload.

### Mitochondrial Characteristics

In the present study, mtDNA copy number tended to decrease (*P* = 0.07) in TAC compared to sham on day 28 after operation, but the difference was not significant (Figure 1A). However, a previous study showed that mtDNA copy number decreased in a similar animal model of aortic banding [6]. This discrepancy may be due to the difference in severity of pressure overload between the two studies, judging from the hypertrophy data. Increasing the pressure overload intensity in our model may result in a significant decrease in mtDNA copy number in TAC. In the present study, we used a 26-gauge needle to induce pressure overload, which produced stable hypertrophy but rather mild effect on heart failure. On the other hand, using a 27- or 28-gauge needle as in previous study [13] resulted in higher surgical mortality but produces greater pressure overload in our preliminary experiments.

We found that increasing mtDNA copy by Twinkle overexpression did not affect mitochondrial enzyme activity, which is consistent with a previous report [16]. Furthermore, TAC also did not affect mitochondrial enzyme activity (Figure 1B and C). These results suggest that mitochondrial electron transport complex activity is not directly related to the cardioprotective effect of Twinkle overexpression.

The mechanism by which increased Twinkle expression prevents heart failure under pressure overload condition remains unknown. In this study we showed that Twinkle overexpression prevented cardiac fibrosis *in vivo* and *in vitro* (to be discussed in detail below). We therefore speculate that Twinkle overexpression somehow inhibits cardiac profibrogenic signals. We need to conduct further investigation about the mechanism.

### Cardiac Hypertrophy, Function, and Fibrosis

Twinkle overexpression ameliorated TAC-induced decreases in LV fractional shortening and ejection fraction, as well as increase in LV end-diastolic pressure (Table 1 and Figure 2). These changes were significant although the magnitudes were small. As mentioned earlier, the relatively mild pressure overload produced in our model may partially explain the small amelioration of cardiac dysfunction by Twinkle overexpression. Nevertheless, the significant improvements in cardiac function indicate the benefit of Twinkle overexpression in preventing heart failure. On the other hand, hypertrophic changes (heart weight/body weight, LV weight/body weight and wall thickness) and end-diastolic LV dilatation were comparable between Tg-TAC and WT-TAC (Table 1 and Figure 2). These findings suggest that Twinkle overexpression does not inhibit adaptive remodeling (myocardial hypertrophy) but attenuates maladaptive remodeling (progression of systolic dysfunction) after sustained pressure overload.

Histopathologically Twinkle overexpression attenuated fibrotic changes after TAC operation (Figure 3), and *in vitro* experiment confirmed the inhibition of profibrogenic genes by Twinkle overexpression (Figure 4 and 5). Cardiac fibrosis is a typical morphological change in maladaptive cardiac remodeling in hypertensive heart disease [24]. Both systolic and diastolic cardiac functions correlate with the degree of cardiac fibrosis [25], [26]. Taken together, we speculate the relatively preserved LV function in Tg-TAC may be associated with the amelioration of cardiac fibrosis.

A major limitation of the present study is that we cannot elucidate the mechanism of how increased mtDNA reduces fibrosis in the pressure overload model. We should conduct further investigations to reveal the molecular mechanisms of how Twinkle overexpression or increased mtDNA decreases fibrosis or fibrosis-related signaling.

### Clinical Implication

We speculate that increased mtDNA copy number by Twinkle overexpression is responsible for the cardioprotective effects. Previous studies have proposed various strategies such as resveratrol intake [14], exercise training [27], and caloric restriction [28] to increase mtDNA copy number systematically. We have also reported that exogenously administered recombinant mitochondrial transcription factor A protein increases mtDNA copy number in cardiac myocytes [15]. Increasing mtDNA copy number in clinical situation using these methods would be beneficial for the prevention of heart failure caused by pressure overload. Further investigations, especially in human studies, are anticipated.

### Conclusion

Overexpression of Twinkle helicase ameliorated the progression of LV fibrosis in a mouse pressure overload model. Increasing mtDNA copy number by Twinkle overexpression could be a novel therapeutic strategy for hypertensive heart disease.

## Supporting Information

Figure S1 **The time course of LV fractional shortening after TAC.** The change of LV fractional shortening over time, after TAC operation. Values are mean ± SEM. *; *P*<0.05 vs day 0, **^†^**; *P*<0.05 vs WT-TAC (day 28). FS; fractional shortening.
(TIF)

Figure S2 **mRNA expressions after TAC operation.** A–C. mRNA expression of COL1a (A), COL3a (B), and CTGF (C), 28 days after TAC or sham operation. They were quantified by real-time PCR relative to nuclear genome (HPRT gene). Values are mean ± SEM. Data are presented as ratio to WT-sham.
(TIF)

Figure S3 **Twinkle mRNA expression in siTwnkle.** Rat Twinkle mRNA expression in cultured cardiac fibroblasts were quantified by real-time PCR relative to housekeeping gene (18S gene). Cells were preinfected with AxCAsi-rTwinkle (siTwinkle) or AxCALacZ (LacZ). Values are mean ± SEM. Data are presented as ratio to LacZ. **; *P*<0.01 vs LacZ.
(TIF)
